# Health-Related Quality of Life of Young Addict Women in Mashhad, IR Iran

**DOI:** 10.5812/ijhrba.10296

**Published:** 2013-09-20

**Authors:** Mohammad Khajedaluee, Reza Assadi, Maliheh Dadgar Moghadam

**Affiliations:** 1 School of Medicine, Mashhad University of Medical Sciences, Mashhad, IR Iran

**Keywords:** Young adult, Substance-Related disorders, Quality of Life, Vulnerability

## Abstract

**Background:**

Quality of life (QoL) stands for to the general well-being of the populations in the societies. Quality of life should not be confused with the concept of standard of living, which is based primarily on income. Instead, standard indicators of the QOL include not only wealth and employment, but also built environment, physical and mental health, education, recreation and leisure time and social belonging.

**Objectives:**

This study attempted to evaluate the QoL of the young addicted women compared to non-addict controls.

**Materials and Methods:**

This case-control study was conducted on young addict women aged between 16-25 years old using with BREF-WHOQOL questionnaire. The subgroups included vulnerable addicts, non-vulnerable addicts and healthy controls.

**Results:**

The findings of the current study demonstrated that drug abuse led to reduce health-related QoL in all aspects of health compared with control group.

**Conclusions:**

Health-related QoL was lower in all aspects of health compared with control group.

## 1. Background

In some societies, including our country, addiction is thought to be a masculine phenomenon. Nevertheless, according to the research results, women comprise approximately 10% of addicts in the country. Based on statistics, 50% female prisoners are in prisons in relation to drug abuse (1).

Although there is a significant gender difference in drug and alcohol abuse, but now a days alcohol and drug abuse is increasing in women. Compared to men, women are less likely to use illegal drugs, but when they start to being addicted, they tend to show addiction faster and would experience negative outcomes sooner than men (2).

Compared to other women, addict women suffer more from serious illnesses and non-communicable diseases, such as hepatitis and AIDS. Negative attitudes about addict women are a major obstacle in the course of treatment. However, women are less likely to receive support from families and friends to quit addiction. Treatment programs often lead to unexpected obstacles in women treatment procedure (3).

Drug abuse has a negative impact on the physical, psychological, social, economic and familial aspects of life. Results of research conducted by Karow to evaluate the role of the social and clinical variables in QoL of addicts, showed that personality disorders, interpersonal conflicts within a family and with spouse, and the need for physical and mental disorders treatment was significantly associated with lower QoL (4).

In a study conducted by Tracy and colleagues that have been performed with WHOQOLBREF questionnaire, findings revealed that the QoL of the addicts is less than normal subjects in various domains. The history of injuries/traumas significantly predicted the level of QoL in mental and physical domains (5).

The term Quality of life (QoL) is used to describe the general well-being of the populations. This term is used in vast domains of international development, health and politics. Quality of life should not be confused with the concept of standard of living, which is based primarily on income. Instead, standard indicators of the QOL include not only wealth and employment, but also built environment, physical and mental health, education, recreation and leisure time, and social belonging (6).

## 2. Objectives

The current study attempted to evaluate the QOL of young addict women and comparing it with the non-addict peers, based on aforementioned tool.

## 3. Materials and Methods

This case-control study conducted on young addict aged 16 to 25 in 2011-2012, Mashhad, Iran. The study groups including vulnerable addicts (those who were jailed to any reason), non-vulnerable addicts (without history of being in prison) and healthy women (those attended regional health centers for reasons other than addiction and had no history of addiction and jail). Sample size is calculated based on the mean difference between two groups (based on previous data studies) and (α = 0.05, β = 0.2) which is equivalent to 80 person in each group. 

Sampling method was randomized stratified in two age subgroups of 16-20 and 21-25 (to ensure including subjects below 20 years old) and in this age subgroups considered in all three groups of the study. All of the subjects signed an informed consent form for the participation in the study. All the information concerning their demographic, familial background, smoking, alcohol and pattern of smoking, and alcohol consumption habits were collected.

### 3.1. Inclusion and Exclusion Criteria

Vulnerable addict group included addicted women aged between 16 to 25 years in prison. No vulnerable addict group included addicted women aged between 16 to 25 years without history of being in prison. Control group included women aged between 16 to 25 years without a history of addiction and being in prison.

In this study the QoL of the individuals was evaluated by WHOQOL-BREF questionnaire. This questionnaire assesses the health related QoL in four domains:

•Physical Health Domain

•Psychological Domain

•Social relationship Domain

•Environmental Domain

This questionnaire contains 26 questions, the first two questions assess the QoL and health statuses in general, respectively, and the rest of questions ask about QoL particularly in the four mentioned domains. This tool is very popular and accepted worldwide. It has been translated to Persian and adjusted for this language in several studies, Yousefi et al. (7) and Nejat et al. (8). Data collection was conducted by trained inquirers participated in a training session to obtain the desired skill and be calibrated in their practice.

## 3. 2. Statistical Analysis

After filling the questionnaires for all subjects, the obtained data was coded based on the answer key and for further statistical analysis all information entered to SPSS software version 11.5. The demographic characteristics of the subjects were presented in descriptive statistics. ANOVA test was used for the analysis of the quantitative variables between groups, when the normal distribution was confirmed; otherwise the Kruskall-Wallis test was implemented. In all of the statistical work the significant level was considered less than 0.05.

## 4. Results

The demographic findings of these subjects are presented in Table 1. The evaluation of this data demonstrated that there is significant difference between groups in case of literacy level, occupation, personal and family income, place of living, smoking and alcohol consumption. 

**Table 1.** Demographic Information of the Subjects

	Vulnerable Addict, N = 80	Addict, N = 80	Control, N = 76	P Valu
**Age**	21.1 (3.33)^a^	21.21 (2.39)	20.90 (2.72)	0.75
**Literacy**				0.001
Illiterate	13 (16.25)	3 (3.75)	0	
Primary School	26 (32.5)	18 (22.5)	0	
High School	30 (37.5)	19 (23.75)	12 (15.8)	
High School Diploma	9 (11.25)	36 (45)	30 (39.5)	
Associate Degree	2 (2.5)	4 (5)	11 (14.5)	
Bachelor	0	0	23 (30.3)	
**Occupation**				0.001
Housewife	33 (41.25)	31 (38.75)	13 (17.1)	
Unemployed	24 (30)	20 (25)	10 (13.2)	
Employed	23 (28.75)	29 (36.25)	53 (69.7)	
Personal Income^b^	209.11 (163.73)	67.69 (82.85)	147.04 (189.90)	
Family Income	340.15 (214.03)	616.66 (177.76)	668.37 (457.19)	
**Place of Living**				0.001
Inside the City	65 (81.3)	55 (68.8)	71 (93.4)	
City Suburb	3 (3.8)	20 (25)	0	
County	11 (13.8)	4 (5)	1 (1.3)	
Village	1 (1.3)	1 (1.3)	4 (5.3)	
**Religion**				0.16
Shia	77 (96.3)	76 (95.3)	76 (100)	
Sonni	3 (3.8)	4 (5)	0	
**Marital Status**				0.001
Single	19 (23.8)	38 (47.5)	75 (98.68)	
Married	28 (35)	31 (38.8)	0	
Divorced	31 (38.8)	9 (11.3)	1 (1.31)	
Widow	2 (2.5)	2 (2.5)	0	
**Smoking**	61 (76.3)	68 (86.1)	1 (1.3)	0.001
**Alcohol**	34 (42.5)	13 (16.9)	0	0.001

^a^Based on type of variable, mean and standard deviation or frequency

^b^Income presented based on Tomans (Iran)

**Diagram 1.**
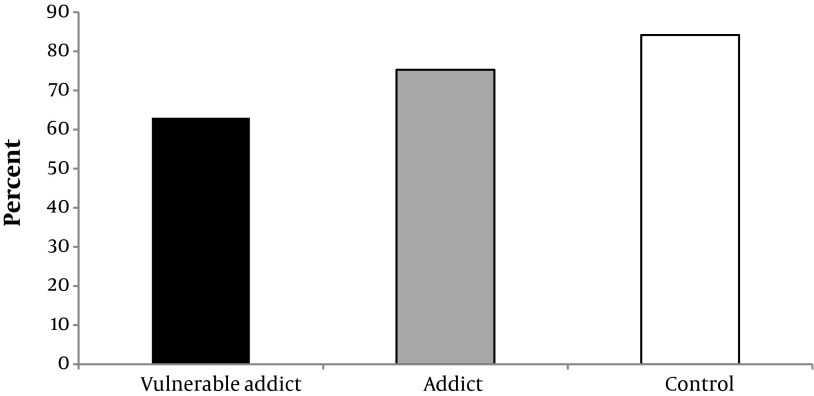
Percent of Physical Health Score

**Diagram 2.**
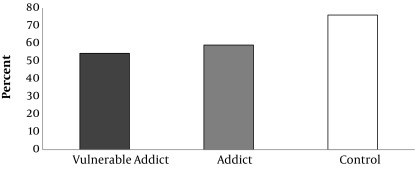
Percent of Mental Health Score

**Diagram 3.**
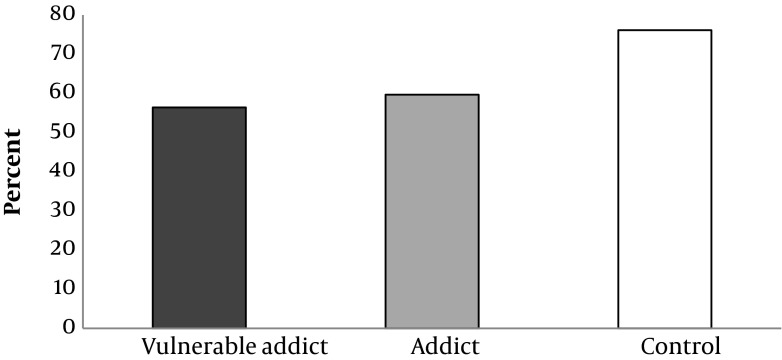
Percent of Social Communication Score Safety

**Diagram 4.**
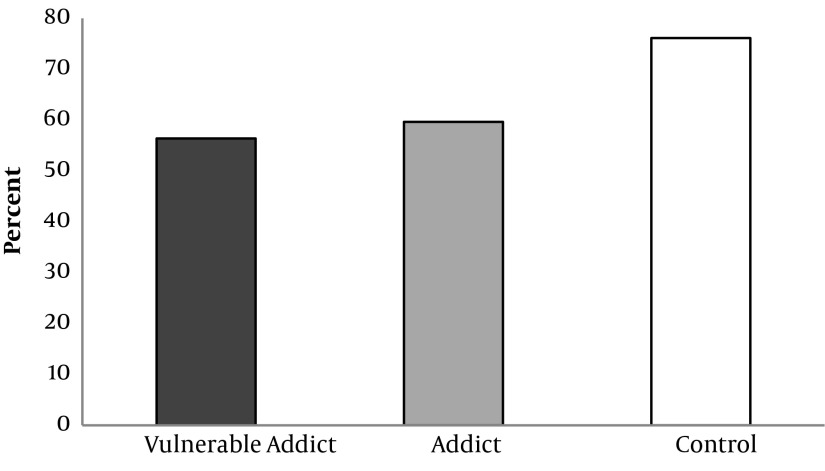
Percent of Environment Health Score

Physical health, mental health, interpersonal communication and environmental safety scores of the studied groups are shown in Diagram 1, 2, 3 and 4, given that the percent of physical health, mental health, interpersonal communication and environmental safety scores were higher in the controls comparing with the other two groups. The mentioned variables in two addict groups were not significantly different. ANOVA test showed a significant difference between three groups in case of physical health, mental health, interpersonal communication and environmental safety scores (P < 0.001).

**Diagram 5.**
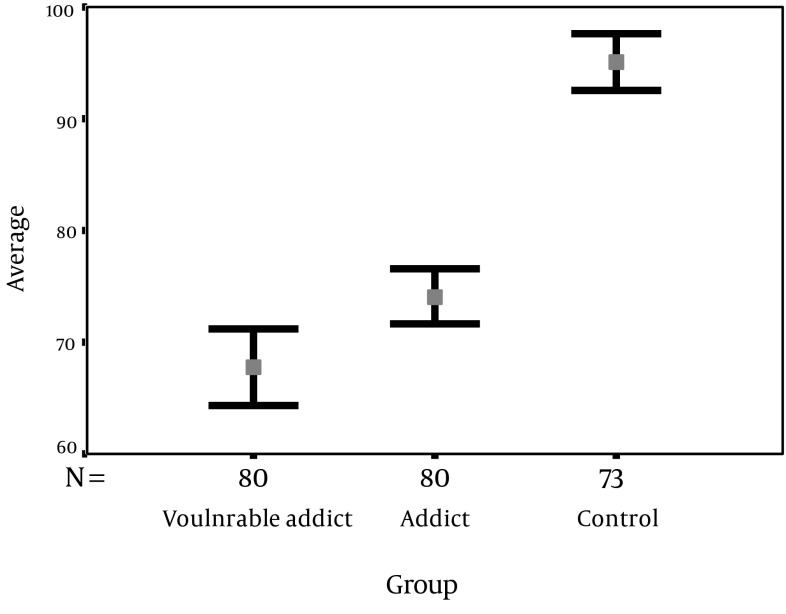
Mean of Total QOL Score in Three Groups

The mean score of total QoL is presented in Diagram 5. The mean score of total QOL in vulnerable addict, addict and healthy subjects was (67.76 ± 15.60), (74.100 ± 11.21) and (95.05 ± 10.69) respectively. Hence the health status of the healthy group is significantly higher than both addict groups, and the addict group higher than the vulnerable ones (P < 0.001).

## 5. Discussion

In the present study the overall score of QoL in general and in four sub-domains was significantly higher in controls compared with addict groups. Further studies showed low quality of life in addict person (9, 10).

In Samad Zadeh and colleagues study which was conducted to evaluate QoL of addict and healthy individuals in City of Tabriz, findings revealed that the addicts had an overall lower QoL compared with healthy individuals. They reported that healthy individuals had a better physical function, general health status, vitality, proper social function and mental health while less physical restriction, emotional conflict and physical pain were reported (11).

In another study conducted by Hojati et al. with the objective of evaluating the QoL of those referred to drug abuse treatment. The findings showed that fifty three percent of the addicts had “to some extent” desirable QoL and just eight percent reported high QoL (12).

Other studies have shown that drug abuse treatment has a positive impact on improving the quality of life for these individuals.

In Lua et al. study, the findings demonstrated that treatment has led to an improvement in the QoL and dramatic changes were observed in QoL of the individuals after treatment (13).

Vang and colleagues studied QoL of Heroin addicts who were under methadone maintenance therapy. Findings showed improvement of QoL in the course of treatment, and further therapy course was predicting for better QoL (14).

This study showed significant differences in factors such as education, employment, household income, personal income and parental educational as a social determinant of health between two addicts and control groups. Addiction affects all aspects of health of women, increases risk of infectious, sexually transmitted and cardiovascular diseases, and induces mental disorders such as depression and anxiety. Deterioration of socioeconomic condition and social dysfunction deepen the mood disorders. These disorders lead to poverty, family breakdown and family rejection that particularly influence psychological and social communication statuses and subsequently QoL of the sufferer. 

The subjects of this study has been women aged between 16-25 years old, which is in active duration of life and the deterioration of QoL will affect their personal and social development as well as roles.

All of the questionnaires in this study were filled anonymously and the subjects were ensured that their confidentiality will be protected and will not be disclosed in any circumstance. All subjects signed and informed consent form for participation in this study.
